# Consistent change propagation within models

**DOI:** 10.1007/s10270-020-00823-4

**Published:** 2020-08-25

**Authors:** Roland Kretschmer, Djamel Eddine Khelladi, Roberto Erick Lopez-Herrejon, Alexander Egyed

**Affiliations:** 1 Institute for Software Systems Engineering, Johannes Kepler University, Linz, Austria; 2 CNRS, Univ. Rennes 1, Rennes, France; 3 D’epartement de g’enie logiciel et des TI, ’ETS - University of Quebec, Quebec, Canada

**Keywords:** Model-driven engineering, Inconsistency repair, Change propagation, Consistency detection

## Abstract

Developers change models with clear intentions—e.g., for refactoring, defects removal, or evolution. However, in doing so, developers are often unaware of the consequences of their changes. Changes to one part of a model may affect other parts of the same model and/or even other models, possibly created and maintained by other developers. The consequences are incomplete changes and with it inconsistencies within or across models. Extensive works exist on detecting and repairing inconsistencies. However, the literature tends to focus on inconsistencies as errors in need of repairs rather than on incomplete changes in need of further propagation. Many changes are non-trivial and require a series of coordinated model changes. As developers start changing the model, intermittent inconsistencies arise with other parts of the model that developers have not yet changed. These inconsistencies are cues for incomplete change propagation. Resolving these inconsistencies should be done in a manner that is consistent with the original changes. We speak of consistent change propagation. This paper leverages classical inconsistency repair mechanisms to explore the vast search space of change propagation. Our approach not only suggests changes to repair a given inconsistency but also changes to repair inconsistencies caused by the aforementioned repair. In doing so, our approach follows the developer’s intent where subsequent changes may not contradict or backtrack earlier changes. We argue that consistent change propagation is essential for effective model-driven engineering. Our approach and its tool implementation were empirically assessed on 18 case studies from industry, academia, and GitHub to demonstrate its feasibility and scalability. A comparison with two versioned models shows that our approach identifies actual repair sequences that developers had chosen. Furthermore, an experiment involving 22 participants shows that our change propagation approach meets the workflow of how developers handle changes by always computing the sequence of repairs resulting from the change propagation.

## Introduction

The benefits of *model-driven engineering (MDE)* hinge on the assumption that models remain consistent. This is obviously a problem during evolution when changes happen. Avoiding or repairing inconsistencies is important, because inconsistencies often cause subsequent errors if developers do not recognize them in a timely manner. Moreover, if models are inconsistent, all automation around them is untrustworthy and likely causes further errors. Therefore, inconsistencies must not only be detected but ultimately be repaired [5, 9, 51].

Not surprisingly, inconsistency detection and repair has received considerable attention from the scientific community [14, 32, 42, 49].

However, not only wrong but also incomplete model changes may cause inconsistencies. That is, a change in a part of the model that was not carried through to other parts of the model causes inconsistencies. Those inconsistencies are cues for missing changes and not for (earlier) erroneous changes. This paper focuses on these incomplete changes. We discuss how to use inconsistencies as guides for completing changes (i.e., for consistent change propagation) by (1) suggesting changes to (not yet modified) parts of the model that repair the given inconsistencies, (2) systematically exploring further changes that repair inconsistencies caused by the changes in (1), and (3) by ensuring that the resulting sequence of changes is faithful to the initial, incomplete changes the developer made (which convey the developers’ intention). We speak of consistent change propagation.

As an example, imagine that a message is passed among two components and the name of this message is inconsistent, because there is no declaration with such a name. One possible repair of this inconsistency is to change the message name. As such, the message name could be changed to the name of any declaration. However, another possible repair is to change the name of one of the declarations. Both are valid repairs but which repair is in fact the desired one? This depends on the developer’s intent. In fact, by considering the original developer’s change that caused the message to become inconsistent, we could infer the intent. For example, if the developer initially renamed the message which caused the inconsistency, then the likely intent was to rename the declaration or add a new declaration. It would be contradictory to the developer’s intent to rename the message once again, even though doing so would be a valid repair.

The basic idea governing consistent change propagation is to treat earlier developer changes as correct modifications of the model. The inconsistencies caused by these changes should then be repaired such that they do not contradict the developer and/or earlier repairs. Consistent change propagation cannot be solved adequately if one merely investigates one change at a time without also considering subsequent changes therefore.

This paper introduces a method and corresponding implementation that explores the propagation of initial developer changes by systematically trying alternatives for repairing arising inconsistencies. This is done by computing a model state tree for each developer change, where every model state represents the application of exactly one repair. This allows us to track every effect the repair has on the model and provide guidance on how a developer can propagate the performed change through affected parts of the model. The result is not a single repair but rather alternative sequences of changes.

We evaluated the performance and usability of our approach on 18 case studies consisting of inconsistent models. Two of those models were taken from GitHub. Among the 176 changes, we were able to reach a consistent model state within five repairs on average for every developer change. We further conducted an experiment with 22 students. The experiment helped us to further assess the usefulness of our consistent change propagation approach, by investigating whether our approach can reproduce the repair sequences the students have applied to the model. We were able to compute all repair sequences the students applied to the models to propagate changes. On average our approach is able to provide all model state trees and repair plans within 3 s over all used models. In cases where models have hundreds of possible repairs to propagate changes, we sorted them by the number of necessary repair steps from shortest to longest repair sequences, i.e., "fewer is better" is often considered a desirable sorting strategy.

**Fig. 1:**
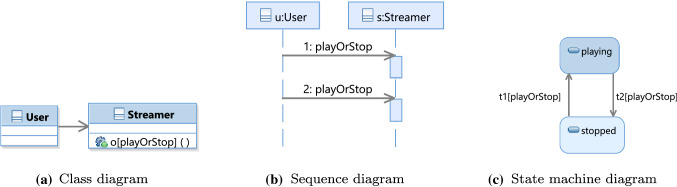
UML model snippets of VOD system

**Table 1:** Consistency rules

Consistency Rule 1 (CR1)—Every message has to have a corresponding operation in the lifeline’s class.	
Consistency Rule 2 (CR2)—Every transition has to have a corresponding operation in the state machine diagram’s class.	
Consistency Rule 3 (CR3)—Every transition has to have a corresponding message in the lifeline.	

## Running example

To illustrate our approach, we use a *video on demand* (VOD) system which is based on a client-server architecture taken from Egyed [6]. Figure 1 depicts example snippets of three different UML diagram types of this system: a class diagram, a sequence diagram, and a state machine diagram.

In the class diagram in Fig. 1a, class User initiates the process of playing or stopping a stream by calling operation playOrStop on class Streamer. Class Streamer handles the user interaction, e.g., receiving user input and visualizing streams. The sequence diagram in Fig. 1b describes the operation where a user plays or stops a specific stream by calling operation playOrStop on an instance of class Streamer, which then initiates the playback of a movie or stops the playback. The state machine diagram describes the possible states of class Streamer. When a Streamer is in the state stopped and a user changes the state by calling playOrStop then the state machine transitions to the state playing (the reverse happens if playOrStop happens again). To distinguish those two transitions in the text, we will annotate them with t1[playOrStop] for the transition from state stopped to playing, and t2[playOrStop] for the transition from state playing to stopped.

In this paper, we use the *Object Constraint Language (OCL)* [37], a declarative language based on first order logic, to define our *consistency rules* for UML models. Table 1 shows three examples of consistency rules (CRs). *Consistency rules* define specific constraints that must hold in software models. These constraints express relations among model elements that can range from well-formedness to very domain-specific ones for non-functional properties such as maintainability or usability [42]. CR1 ensures that all messages in a lifeline have an equally named operation in their corresponding class. CR2 ensures that every transition in a state machine diagram has an equally named operation in its corresponding class. CR3 checks that every transition in a state machine diagram has an equally named message in its corresponding lifeline.

The focus of this paper is supporting developers in propagating changes. Hence, the initial change is made by the developer. For example, let us imagine that a developer wants to split the playOrStop transition into two separate transitions play and stop. The developer may initiate this by first renaming transition t1[playOrStop] to t1[play]. However, doing so causes two inconsistencies:

I1: **Violation of CR2.** There is no operation named play in class Streamer for the new transition play.
I2: **Violation of CR3.** There is no incoming message play to lifeline s:Streamer for transition play.

Our approach assumes that the initial developer change was performed with intent (i.e., the software engineer wants to evolve the state machine diagram in this manner). The resulting inconsistencies need to be repaired with respect to this intent.

It is easy to see that these inconsistencies imply incomplete changes. Splitting playOrStop requires additional changes in the sequence and class diagrams. Without automated support, the developer may be unaware of this problem. Even if the developer knows about the inconsistencies then she may not know which further changes are needed to resolve the inconsistencies. It is important to note that there are many alternatives to propagate a change throughout the whole model. A repair mechanism that does not understand the developer’s intent would for instance, suggest to repair I1 by either (1) renaming transition play to playOrStop, (2) adding an operation with name play to the model or (3) renaming the existing operation playOrStop. Obviously, repair 1 is valid but contradictory to the developer’s intent. Repairs 2 and 3 appear reasonable but what are their effects? Are they equal? For example, repair 3 would resolve I1 but it would introduce further inconsistencies. Moreover, recall that the initial developer change caused two inconsistencies.

Starting to propagate the developer change by fixing I1 is one possibility. Another possibility is to propagate it by fixing I2, for example renaming message 1:playOrStop (in Fig. 1b) to play, but that causes a new inconsistency I3.

I3: **Violation of CR1.** There is no operation named play in class Streamer for message play.

To fix I3, we further propagate the above repair by adding a new operation play to class Streamer, which fixes I3 and also I1. After this step, the model is consistent and the developer’s change from the state machine diagram has been propagated to all corresponding diagrams. While the repairing of I2 with the consequence I3 has the beneficiary side effect of also repairing of I1, the opposite could be true also: the repair of I2 with I3 could contradict I1.

Please note that instead of adding an operation named play to class Streamer it would be also possible to rename operation playOrStop to play, which propagates the developer’s change, but creates two new inconsistencies: no operation for message 2:playOrStop and no operation for transition t2[playOrStop] in class Streamer. To fix those two inconsistencies we can add operation playOrStop to class Streamer, which would result in the same consistent model state as shown above (i.e., presence of operations play and playOrStop). This is an alternative sequence of propagating the change, but it needs one additional action to fix the new inconsistencies.

As another example after play has been propagated, the software engineer changes the transition t2[playOrStop] in the state machine diagram to stop. This again causes two inconsistencies:

I4: **Violation of CR2.** There is no operation named stop in class Streamer for transition stop.
I5: **Violation of CR3.** There is no incoming message stop to lifeline s:Streamer for transition stop.

This time we start propagating the developer’s change to class Streamer by fixing I4. Again we have two possibilities: either we add operation stop to class Streamer or we rename operation playOrStop. Renaming the already existing play should not be done here, since it has been propagated beforehand, and would undo this propagation. We can rename operation playOrStop to stop and then rename message 2:playOrStop also to stop. Thus, class Streamer then only contains two operations play and stop and the model is consistent. However, as an alternative, we can also add an operation stop to Streamer, the result would be three operations (e.g., play, stop, playOrStop) in class Streamer after reaching a consistent model state.

All these repair sequences are alternative change propagations that are a consequence of the initial developer change to reach a consistent model state again. As this example illustrates, repairs should follow the developers’ intent, and there are alternative possible repair sequences, for satisfying this intent. Invalid repair sequences do not resolve all inconsistencies caused or may change the developer’s intent (e.g., by overwriting the developer change). Our approach propagates the change of the developer by computing relevant repairs only, and not all possible repairs in contrast to the existing work in literature.

## Background

This section provides definitions and examples of the most important terms for a proper understanding of this paper, as well as an explanation of the consistency checking mechanism.

### Definitions

#### Definition 1

**Model**. A model ${\mathbb {M}}$ consists of model elements $(me\in {\mathbb {M}})$ where model elements can have properties py. A property of a model element is referred to by element dot (.) property name, e.g., "Streamer.name". A diagram is simply a subset of model elements from the model.

#### Definition 2

**Consistency Rule** A consistency rule is a condition defined for a context. For example, CR1 from Sect. 2 defines a condition that every message has to satisfy. The condition itself is a hierarchically ordered (tree-based) set of expressions, where the root expression corresponds to the condition as a whole and its subexpressions correspond to parts of the condition.

An expression identifies an operation, has a single parent and one or more children and values to be validated (se2v). Recall that CR1 has two parts: an exists expression(->exists(...)) and an equals expression (=). The equals expression has two children: self.name and o.name, both are leaf expression. Typically, leaf expressions either access model elements or are constants.

#### Definition 3

**Validation Tree** A consistency rule validated on a specific model element is a validation. For example, there are two messages in Fig. 1b: 1:playOrStop and 2:playOrStop. Hence, there are two validations, one for each message. Each validation checks if a consistency rule’s condition validates to true for its given context. This can be done recursively for every expression/subexpression of a condition. The root expression of a condition is expected to validate to true, however, as earlier work has shown, this expectation may change with subexpressions (e.g., because of negations [42]). A validation tree mirrors the tree structure of the consistency rule condition. However, in case of repetitions (e.g., exists quantifier above) their (sub)tree structures repeat for every iteration. Hence, the validation tree is an exact log of each operation computed during the validation of a condition. As an example, Fig. 3 shows a validation tree for CR1. This validation tree will be explained in detail in the next section.

#### Definition 4

**Scope Element**. A scope element is a model element and its properties (*e*.*p*) accessed during the validation of a consistency rule. A set of scope elements is called a scope. The scope is derived from the various property call expressions of the validation tree.

#### Definition 5

**Repair Action**. A *repair action* defines a change to a model that resolves an inconsistency in part or full (often multiple repair actions are needed to resolve an inconsistency). A repair action contains the model element (me), the property (py) that is affected by the change, the type of operation(op), and a value (v, which can be a model element $v \in {\mathbb {M}}$, or a primitive value $v \in {\mathbb {V}}$) or no value($\varnothing$) applied to the property. The following types of changes are possible: $+$ adds a value, − deletes a value, and $=$ modifies a model element property to a given value. In addition there are the constraining changes: $\ne$, <, >, where respectively a property has to be different than value, less than value, or greater than value. $\mathbb {RA}$ is the set of all possible repair actions.

$$\begin{aligned} \begin{aligned} ra \in \mathbb {RA}&:= \langle me.py, op, v \rangle , op \in \{+, -, =, \ne , <, >\} \end{aligned} \end{aligned}$$

Literature distinguishes between *abstract* and *concrete* repair actions, where an abstract repair action has no concrete value ($v=\varnothing$). In this paper, we only use concrete repair actions, since abstract repair actions cannot be executed and therefore are not suitable for automated consistent change propagation.

As an example the inconsistency I2 discussed in Sect. 2 can be fixed by changing the name of message 1:playOrStop. Expressed as an abstract repair action this leads to: $\langle 1:playOrStop.name, =, \varnothing \rangle$. Note that this abstract repair action is a hint and is not automatically executable yet, because we do not have a specific value for 1:playOrStop.name.

To fix I2 (from Sect. 2) we can use the following concrete repair action: $\langle 1:playOrStop.name, =, "play" \rangle$, which renames 1:playOrStop to "play". Note that it might be necessary to change multiple scope elements at once to fix an inconsistency. For that purpose, we define groups of repair actions as follows.

#### Definition 6

**Repair**. A *repair* is a non empty collection of repair actions (*ra*) that fixes a specific inconsistency (*i* from the set of all possible inconsistencies ${\mathbb {I}}$). This set *ras* (repair actions) may contain both repair actions which can be abstract (*isAbstract*(*ra*)) and/or concrete ($\lnot isAbstract(ra)$).

$$\begin{aligned} \begin{aligned} \langle i \in {\mathbb {I}}, ras \subseteq \mathbb {RA}_i \rangle \end{aligned} \end{aligned}$$

Furthermore we define the term *abstract repair* which states that the set of repair actions contains at least one abstract repair action ($ra \subseteq \mathbb {RA}_i | (\exists x \in \mathbb {RA}|isAbstract(x)$), and we also define the term *concrete repair* which exclusively contains concrete repair actions ($ras \subseteq \mathbb {RA}_i | (\forall ra \in ras|\lnot isAbstract(x)$). Please note that only a concrete repair is able to fix an inconsistency automatically.

As an example, I2 from Sect. 2 can be fixed by changing the name of message 1:playOrStop. Expressed as a concrete repair this leads to: $\langle I2, \{ \langle 1:playOrStop.name, =, "play" \} \rangle$. Basically each correct value leads to a concrete repair.

#### Definition 7

**Developer Change** A developer change is a single intentional modification of a model element performed by human. A developer change is similar to a repair action (i.e., it affects a model element, one of its properties, has an operation $+, -, =$, and always a concrete value v); however, it may create or repair new inconsistencies, or it may have no effect on the model’s consistency.

$$\begin{aligned} \langle me.py \in {\mathbb {M}}, op, v \rangle , op \in \{+, -, =\} \end{aligned}$$

As an example, consider the developer change from Sect. 2 where a developer renamed transition t1[playOrStop] to play. Expressed as tuple this leads to: $\langle t1[playOrStop].name, =, "play"\rangle$

#### Definition 8

**Model State** A model state represents an applied change to the model *r* (through the execution of a repair) leading to a changed set of inconsistencies (*i*), where at least one inconsistency is repaired, and possibly new inconsistencies are caused. Every model state has a preceding model state (one change applied before) called parent *p* and a set of multiple succeeding model states (multiple repairs applied after) called children *c* forming a *model state tree*.

$$\begin{aligned} \langle r \in {\mathbb {R}}, p \in \mathbb {MS}, c \subseteq \mathbb {MS}, i \in {\mathbb {I}}\rangle \end{aligned}$$

As an example, consider the change of renaming message 1:playOrStop to play from Sect. 2. Expressed as tuple this leads to:

$$\begin{aligned} \Big \langle \big \langle I2, \{ \langle 1:playOrStop.name, =, "play"\rangle \} \big \rangle , \varnothing , \varnothing , \{I1, I3\} \Big \rangle \end{aligned}$$

#### Definition 9

**Repair Sequence** A repair sequence represents a list of repairs, which (when executed in order) lead to a final model state where the initial developer’s change has been propagated. The set $\mathbb {DC}$ represent all possible developer changes in the model.

$$\begin{aligned} \begin{aligned} \langle u \in \mathbb {DC}, \langle r_1 \in {\mathbb {R}}, \dots ,&r_n \in {\mathbb {R}} \rangle \rangle \end{aligned} \end{aligned}$$

For instance, a repair sequence from Sect. 2 is:

$$\langle \langle t1[playOrStop].name, =, "play" \rangle , \langle \langle I2, \{ \langle 1:playOrStop.name, =, "play" \}\rangle , \langle I3, \{ \langle Streamer.operations, +, play \} \rangle \rangle \rangle $$

**Fig. 2:**
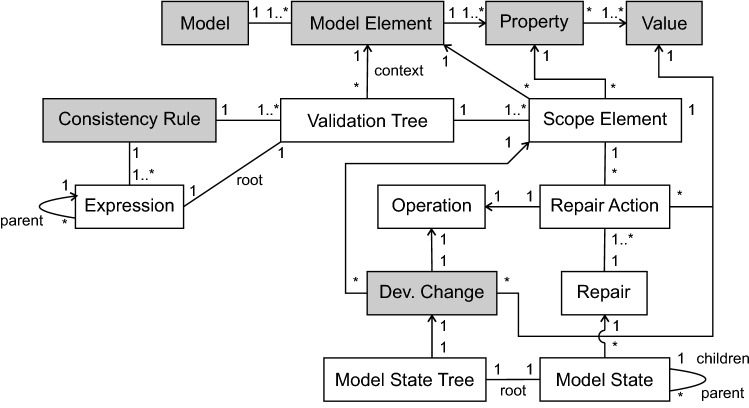
UML class diagram for the definitions

Which first applies the developer change (rename t1[playOrStop] to play) then message 1:playOrStop.name to play and finally adds an operation play to class Streamer.

#### Definition 10

**Model State Tree** A model state tree represents sequences of repairs that propagate a developer change to a consistent model state. The repair sequences are implicitly represented by the hierarchy of the model states. A model tree’s origin is always a developer change *dc*, it consists of at least on model state and an arbitrary amount of original inconsistencies *oi* (e.g., inconsistencies present before the developer change has been applied to the model). Please note that we do not consider those inconsistencies, since they are not created by the developer change. We only use consistent propagation for the new caused inconsistencies.

$$\begin{aligned} \langle dc \in \mathbb {DC}, ms \subseteq \mathbb {MS}, oi \subset {\mathbb {I}} \rangle \end{aligned}$$

### Relations of the defined terms

For a better understanding of how our defined terms are related to each other, we give an overview in Fig. 2. This figure shows a UML class diagram of the definitions from the previous section depicted as classes (without attributes) and their associations. For instance a Validation Tree has exactly one Model Element as context element, depicted as association from Validation Tree to Model Element with the name context. In turn, one Model Element can be used by multiple Validation Trees as their context element.

The boxes highlighted in grey are the foundation of our approach and are provided by an engineer.

### Consistency checking

Consistency checking is a well-covered topic in literature. In this section, we explain one such approach on a simple example. To illustrate the consistency checking mechanism, we use CR1 from Sect. 2.

**Fig. 3:**
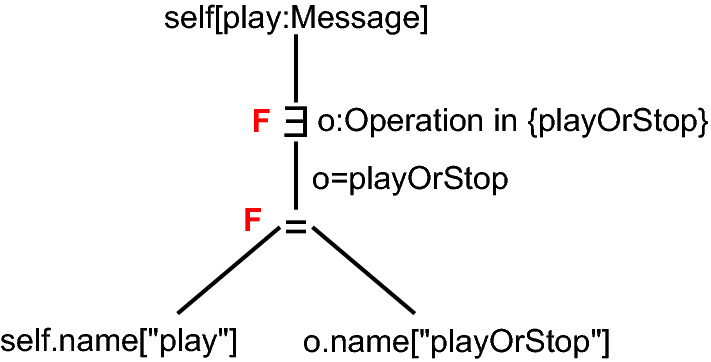
Validation Tree for CR1



Figure 3 shows the instantiation of CR1 for the message play. The root expression represents the model element, for which the consistency rule is instantiated self[play:Message]. The next expression is an exists expression ($\exists$) where at least one of its children has to fulfill the condition defined in CR1 (self.name = o.name).

At the exists expression we create one subtree for operation playOrStop, which is in the corresponding class Streamer of the message play. Remember that message play is the modification performed by a developer (i.e., the developer change) from Sect. 2. If there would be more operations, every operation results in its own subtree in Fig. 3. This subtree represents an equals expression ($=$) which compares the values returned by their children for equality. The comparison is between the message’s name play and the operation’s name playOrStop.

The scope elements of those expressions in the validation tree in Fig. 3 are [play:Message].name, [playOrStop:Operation].name, Streamer:Class and s:Lifeline. Together those four scope elements form the scope for CR1.

In the validation tree, the root expression is expected to validate to true (i.e., consistent) and so its children expressions. For example, the $\exists$ and $=$ expressions in Fig. 3 are also expected to validate to true. If the root expression validates to false, then we detect an inconsistency. To compute the validation result of a validation tree, we start from the leafs (bottom) and start computing the validation result of the subexpressions (parent nodes) and continue this process until the root expression.

In Fig. 3, since the name "play" is unequal to "playOrStop", the subtrees’ validation result is false (denoted with a red F). At the exists expression ($\exists$), there has to exist at least one subtree in its children with the validated result true, but in this example the subtree validates to false. Thus, the exists expression validates to false, which is the same validation result of the root expression. This is how the inconsistency I1 from Sect. 2 is detected in the model based on its validation tree.

### Repair generation

In the previous subsection, we explained how inconsistencies in models are detected. In this section, we introduce, why we can repair arising inconsistencies in software models. Before computing the repairs, we first need to identify the cause of an inconsistency.

Take again the inconsistency I1. To identify the *cause* for I1 we take the *scope* from the previous section $scope=\{$[play:Message].name, [playOrStop:Operation].name, Streamer:Class, s:Lifeline$\}$. We then check for every scope element, if it is part of a violated expression, i.e., validation result $\ne$ expected result (validation result of those expressions is false in Fig. 3), we add it to the *cause* of the inconsistency. The *cause* of our example shown in Fig. 3 is $cause=\{$[play:Message].name, [playOrStop:Operation].name, Streamer.operations $\}$.

To generate repair actions, we iterate over every scope element in the *cause* and look for every violated expression were the scope element is used. We then generate a change (i.e., repair action) so that the direct violated expression is validated. For instance, the scope element [playOrStop:Operation].name is used in the violated $=$ expression in the right-hand side of the validation tree shown in Fig. 3. Based on this expression we know that the equals condition $=$ is not fulfilled, since name playOrStop is unequal to name play. Therefore, the repair action for the scope element playorstop would be to rename it to play $\langle playOrStop.name, \odot , "play" \rangle$, which leads to the repair $\langle I1, \{\langle playOrStop.name, \odot , "play" \rangle \}\rangle$ that fixes I1 when executed.

However, changing the name of operation playOrStop to play is not the only valid repair for fixing I1. Based on the $=$ expression in Fig. 3, another repair can be generated for the scope element play which is to rename message play back to playOrStop $\langle I1, \{\langle play.name, =, "playOrStop" \rangle \}\rangle$. This repair also fixes I1, however it would not make sense to rename play to playOrStop as it would undo the initial developer change. For a more detailed explanation of the repair generation mechanism, please refer to [40].

## Change propagation approach

This section presents our automated approach to consistently propagate a developer change to other parts of the model. The resulting sequences of repairs guide the developer towards a consistent model state containing the information provided by the initial developer change. First we give a general overview, then we describe how we perform the change propagation.

**Fig. 4:**
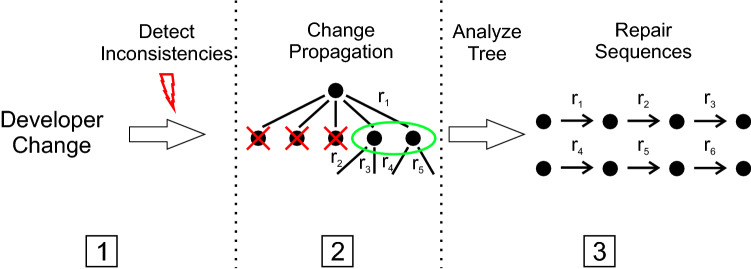
Overview of our approach

Figure 4 shows the basic workflow of our approach, which consists of the following three stages.

The *first stage* () applies the developer change to the model and identifies arising inconsistencies caused by this change. To detect those inconsistencies, consistency rules written in OCL are applied to the model via a consistency checking approach (e.g., [41, 42, 54]). Those approaches come up with abstract repairs, which can be transformed to concrete repairs by using [13, 23].

The *second stage* () creates a model state tree for each of the initial developer changes causing inconsistencies. The model state tree explores new model states by repairing arising inconsistencies, where every repair leads to a new model state.

The *third stage* () analyzes the model state trees generated in the previous stage. This stage looks for final model states that are consistent (i.e., the leafs) and collects every repair applied from the root to those model states. Of course, there can be many possibilities of propagating a change resulting in a consistent model state. In the end the developer has to choose which one of them satisfies her needs. To help developers in choosing a repair sequence, we rank the repair sequences (from shortest to longest) based on the number of repairs.

### Stage 1: Initial model state tree


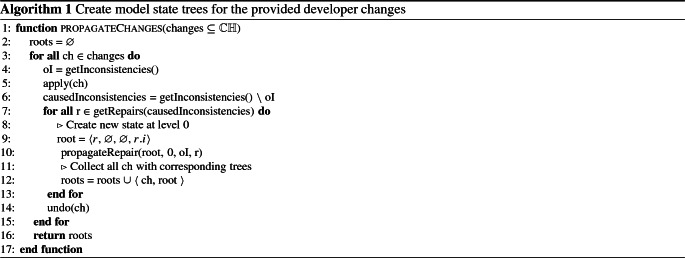


The first stage () applies the provided developer changes sequentially (one after the other) through the impacted model elements and diagrams. After all developer changes have been applied, each developer change has a corresponding model state tree as defined in Sect. 3. Those model state trees can then be used to provide repair sequences for each developer change. Algorithm 1 illustrates the first stage () of the developer change application.

The function propagateChanges takes a set of developer changes and returns a set of model state trees for each of them. From Line 3 to Line 6 we iterate over every provided developer change ch in changes and check the consistency of the model before we apply ch. This prevents the interference from already existing inconsistencies in the model. We then apply ch and check the model for the caused inconsistencies (caused by ch) (causedInconsistencies) by removing the already existing inconsistencies from the new set ($\setminus$ is the set difference operation). Inconsistencies present before the application of the developer change are not considered for change propagation, since they are not caused by the developer change. However, as they are inevitably caused by previous changes, they could naturally be repaired with our current work by considering their causing changes too. For the sake of simplicity, we focus on new inconsistencies caused by developer changes.

From Line 7 to Line 16 we first iterate over every possible repair r from the caused inconsistencies (r $\in$ getRepairs(causedInconsistencies)).

The function getRepairs() generates repairs based on validation trees (see Sect. 3.4), which originate from the provided consistency rules. We use our previous work [23, 40, 42] to compute repairs. However, our change propagation approach is generic and can work with repairs computed by any of the existing related works.

After that for each repair, we create a new model state at level 0 with the original inconsistencies oI (every sub model state needs to know them, to avoid propagating their repairs), no parent, no children (at this moment) and the repair r itself. We then start the propagation of the current developer change ch for every subsequent repair r by calling propagateRepair() (stage ). After the propagation of one developer change ch in changes, we undo the change (calling undo(ch)) so that we can propagate the next change without interference from the previous one. After all changes have been propagated we return their model state trees, so they can be analyzed afterwards (stage ). Stages  and  are discussed in the next sections.

As an example to better illustrate the first stage (), consider the model shown in Fig. 1 and the developer change $\langle t1[playOrStop].name, =, "play" \rangle$ from Sect. 2. In this example, we have one developer change to propagate ch=$\langle t1[playOrStop]. name, =, "play" \rangle$. Before we apply ch there are no inconsistencies in the model (oI = $\varnothing$). After the application of ch, the two inconsistencies I1 and I2 from Sect. 2 are detected (causedInconsistencies = { I1, I2 }). The possible repairs for those two inconsistencies can be seen in Table 2.

**Table 2:** Repairs for I1, I2, I3, I4 and I5

Abbreviation	Repairs
R1	$\langle I1, \{\langle o[playOrStop].name, =, play \rangle \} \rangle$
R2	$\langle I1, \{\langle Streamer.operations, +, play \rangle \} \rangle$
R3	$\langle I2, \{\langle 1:playOrStop.name, =, play \rangle \} \rangle$
R4	$\langle I2, \{\langle 2:playOrStop.name, =, play \rangle \} \rangle$
R5	$\langle I2, \{\langle s:Streamer.messages, +, play \rangle \} \rangle$
R6	$\langle I3, \{\langle o[playOrStop].name, =, play \rangle \} \rangle$
R7	$\langle I3, \{\langle Streamer.operations, +, play \rangle \} \rangle$
R8	$\langle I4, \{\langle Streamer.operations, +, playOrStop \rangle \} \rangle$
R9	$\langle I5, \{\langle Streamer.operations, +, playOrStop \rangle \} \rangle$

**Fig. 5:**
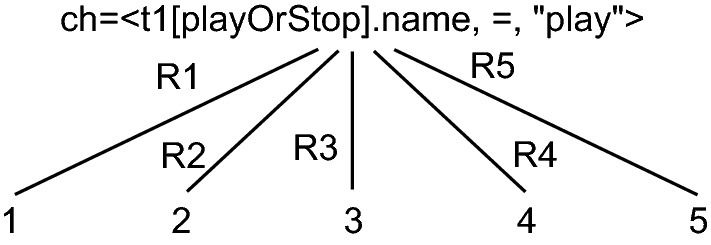
Initial model state tree for a developer change

Of course another additional repair for both inconsistencies would be to rename transition play back to playOrStop, but this would undo the developer change and performs no propagation. Hence, it is not considered as relevant repair option. After those first repairs have been found, we get an initial propagation tree with only one level, as shown in Fig. 5. This tree depicts all possible model states (labeled with 1 to 5) after applying the repairs shown in Table 2. All the shown repairs have the same structure, where either a value is modified ($=$) or an element added ($+$). For instance, R1 renames operation o[playOrStop] to play, and R2 adds an operation play to class Streamer.

### Stage 2: Model state propagation

The next step is to further propagate those repairs towards a consistent model, since those five repairs cause subsequent inconsistencies. The second stage ( ) depicts this process shown in Algorithm 2.
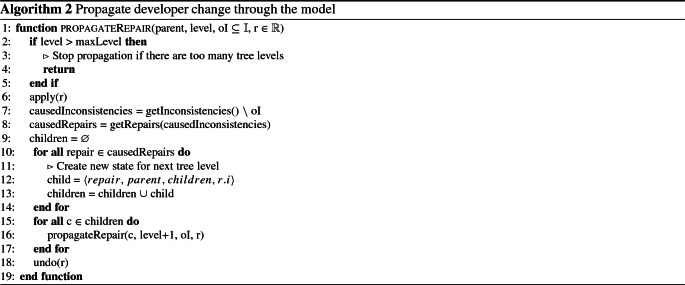

From Line 2 to Line 6, we check the current model state reached the limit of the current model state tree by checking its level with the maximum admissible level (maxLevel). This is done to skip propagation in cases where it takes too long to reach a consistent model state (i.e., to much inconsistencies caused by subsequent repairs). It also aims to skip cycles of repairs that would lead to an endless recursion in the tree. We then apply the repair to the model to possibly get new inconsistencies (apply(r)). From Line 7 to Line 14 we retrieve new repairs from the inconsistencies caused by the application of repair r (getRepairs(causedInconsistencies)) with respect to the original inconsistencies oI. Repairs from oI are not considered for the propagation, since they have not been created by the initial developer change. If there are no new repairs, our algorithm has found a consistent model state and the developer change has been propagated successfully. If we detected new repairs (causedRepairs not empty) we create a new model state for each repair at the next level (level + 1) and add this new state to the children of the current state (children = children $\cup$ child). Then, we continue propagating the change for every child recursively to the next level (propagateRepair(c,level+1,oI,r). After every recursive step, we undo (undo(r)) the applied repair to restore the parent model state and to explore another branch in the tree.

**Fig. 6:**
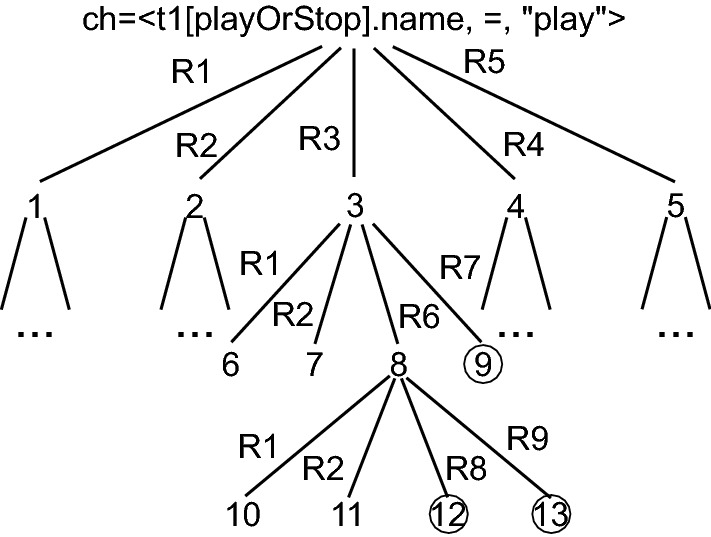
Model state tree for a developer change

After running the algorithm 2, the model state tree in Fig. 5 is augmented by propagating the subsequent repairs R1-R5 resulting in the model state tree shown in Fig. 6. Take for example R3, as mentioned in Sect. 2, R3 repairs I2 but also creates a new inconsistency I3 which can be repaired with the repairs R1,R2, R6, and R7.

For simplicity, let us focus on the propagation of two repairs R6 and R7 in this example. First we select R7 and propagate this to the next model state 9 (c.propagateRepair(3)). After we apply R7 in model state 9 (apply(R7)) we detect no more inconsistencies, and therefore no more repairs. So we undo R7 and return to model state 3. Model state 9 now represents a consistent model state (marked with a circle) in which the initial developer’s change has been propagated successfully. Back in model state 3 we now select R6 (the next repair in collection children). We then continue propagating the change to the next model state 8. After we applied R6 we create two new inconsistencies I4 and I5 (no operation playOrStop for message 2:playOrStop and transition t2[playOrStop]) with the corresponding repairs shown in Table 2. In model state 8, we now propagate R8 and R9 to the consistent model states 12 and 13.

Model states 9, 12 and 13 represent now model states in which the initial developer’s change $\langle t1[playOrStop].name, =, "play" \rangle$ has been propagated and all related inconsistencies have been fixed. The second developer change $\langle t2[playOrStop].name, =, "stop" \rangle$ (from Sect. 2) is propagated following the same principles as shown in this example.

### Stage 3: Analyze model state tree

In stage three ( ), we analyze the model state trees that have been computed in stage , to derive the repair sequences which propagate the developer change and lead to consistent model states. This is performed with a simple depth first search. For example, from the model state tree shown in Fig. 6, after analyzing the subtree R3, we get three repair sequences (repairs from Table 2) which propagate successfully the developer change:

$$\begin{aligned} \begin{aligned} \langle t1[playOrStop].name, =, "play" \rangle&: \langle R3, R7 \rangle \\ \langle t1[playOrStop].name, =, "play" \rangle&: \langle R3, R6, R8 \rangle \\ \langle t1[playOrStop].name, =, "play" \rangle&: \langle R3, R6, R9 \rangle \end{aligned} \end{aligned}$$

**Table 3:** Model information

Model name	#Model elements	#Changes	Sum repaired Incon.	Source
pro11	284	2	4	GitHub${}^\mathrm{a}$
fullAdder	992	1	5	GitHub${}^\mathrm{b}$
VOD	467	3	8	Academia
Curriculum Planer	868	26	99	Academia
eBullition	1346	5	22	Industry
MVC	1410	2	9	Industry
Inventory	1422	17	81	Industry
Tele	1471	7	21	Industry
Course System	1620	29	91	Academia
Vacation System	1805	9	21	Industry
Home Control System	1882	10	89	Industry
DESI	2056	3	3	Industry
Micro	2346	5	9	Industry
iTalks	2462	9	58	Industry
Hotel Management	2790	3	15	Academia
Calendarium	3263	20	50	Academia
Dice	4485	16	30	Industry
dSpace	8859	5	5	Industry
experiment	210	4	21	Academia

${}^\mathrm{a}$https://github.com/11TCLC-DA-CNPM/doan-cnpm-quanly-ban-dien-thoai-pro11tclc/

${}^\mathrm{b}$https://github.com/acdoorn/design-patterns/tree/master/diagrams/

We also rank those sequences based on the amount of repairs, which makes the selection process easier for the developer from shortest to longest. In the end, the developer has to decide which one of those repair sequences fits the most her needs and requirements.

## Evaluation

We evaluate our approach by assessing its correctness, scalability and usefulness. For the evaluation, we applied 20 consistency rules to 18 models taken from three different sources: academia (VOD, Curriculum Planer, Course System, Hotel Management, Calndarium), industry (eBullition, MVC, Inventory, Tele, Vacation System, Home Control System, DESI, Micro, iTalks, Dice, dSpace) and GitHub (Pro11, fullAdder) [11], and one model used in an experiment for this paper. The domains of the models range from control of a microwave oven to a model view controller of software and an inventory storage management system. Two of these models from GitHub have two versions each, where version one had inconsistencies that had been fixed in version two by a developer. This further allowed us to assess the quality of our approach and the relevance of our repairs, i.e., whether the manually applied repairs by the developers could be replicated by our approach. The model sizes ranged from 300 to 8800 model elements, the number of applied changes from 1 to 29 and the sum of all repaired inconsistencies during the propagation of all changes from 4 to 99. Table 3 shows those details per model. Note that our implementation has a compilation module integrated to check the syntactical correctness of the OCL consistency rules. The data set is archived in the FigShare platform^1^ to be used for reproducibility and for comparison purposes. We executed the evaluation on a Windows 10 PC with a Core i7 3.4GHz and 32GB RAM.

### Research questions

In this section, we define three research questions (RQ) to evaluate our approach.

**RQ1:** To what extent is it possible to propagate a developer change based on model consistency information? This aims to investigate if it is feasible to propagate developer changes based on caused subsequent inconsistencies through the model.

**RQ2:** How many consistent model states does our approach find, what is the average length of the found repair sequences, and how much time did it take to compute the model state trees? This aims to investigate the performance and scalability as well as the correctness of our approach, when a developer change has been successfully propagated through the model.

**RQ3:** Does our approach also find repair sequences a developer would have applied to the model? This aims to find out if we can reproduce relevant repair sequences a developer also would have performed to propagate a change when repairing her inconsistencies, thus assessing our approach usefulness.

### Results

#### RQ1

To propagate developer changes, we needed models where developers modified the model, and those modifications led to inconsistencies. Our models (VOD to experiment) are inconsistent, but we do not have the original changes which caused them. However, we can treat their repairs as developer changes that might further cause inconsistencies and thus can be propagated in our approach. First, we use our models from Table 3 with the already existing inconsistencies (present before any applied change). We then analyze those inconsistencies and treat the resulting repairs as developer changes. After all, a repair is similar to a model change with the same implications as a change made by a developer.

Since we convert abstract to concrete repairs based on [23], it may happen that this approach is not able to find concrete repairs for a given abstract repair. In this case, we use other abstract repairs from the same inconsistency. In the cases where there are no concrete repairs for an entire inconsistency, e.g., a completely new class must be added to the model, we cannot propagate the change further. In this case, user intervention might be necessary. However, this was never the case. In fact, we are able to transform for each inconsistency at least some abstract repairs to concrete ones.

**Table 4:** Evaluation results

Model name	#Repair Sequ.	Avg repair Sequ. length	time [s]
pro11	34	3	0.065
fullAdder	29	3	0.075
VOD	60	3	0.076
Curriculum Planer	937	4	25
eBullition	434	6	3
MVC	73	3	0.313
Inventory	4875	13	19
Tele	163	4	7
Course System	2650	6	0.924
Vacation Planer	620	4	0.842
Home Control System	3453	16	583
DESI	10	1	3
Micro	76	2	0.367
iTalks	1248	8	291
Hotel Management	955	21	18
Calendarium	834	4	34
Dice	342	4	0.672
dSpace	18	1	1
experiment	651	7	0.297

We successfully propagated 176 changes, each leading to one propagation tree, in our 18 models and repaired a total amount of 642 inconsistencies during this process leading to hundreds of repair sequences. For instance, we propagated five changes in model Micro while repairing nine caused inconsistencies. This shows the feasibility of our approach that it can indeed successfully propagate changes to consistent model states.

#### RQ2

Additionally, we also analyzed how many consistent model states we were able to detect in all our models. Table 4 shows the results of this analysis. Column #Repair Sequ. shows the total amount of all repair sequences of all propagated changes per model leading to a consistent model state. Please note that we added a limit (i.e., maximum tree level) to the model state trees of 50 repairs, which limits the maximum amount of repairs in a repair sequence to this number. This limit was introduced in cases where the propagation takes too long, resulting in too many repairs. However, we never reached this limit, and the maximum length of all repair sequences was 37 repairs. Each repair sequence represents one possibility to propagate a change to a consistent model state. This shows the correctness of our change propagation which allows developers to reach a consistent model state.

The column Avg Sequ. Length shows the average length of all repair sequences per model. This means that in most cases a developer change can be propagated with one to 21 repairs. Column time shows the average time per model it took to propagate a change to a consistent model state. In most cases, we are able to propagate a change within milliseconds up to 10 minutes.

For example, applying five changes to the model Micro leads to a total amount of 76 repair sequences (15 on average per change) and an average repair sequence length of 2. To detect all 76 repair sequences our approach needed 367ms. Please note that a developer would propagate each change by looking at the computed repair sequences. From those sequences she might choose the one with the least amount of repairs, or one which affects only specific parts of the model (e.g., class diagrams). After that she can choose the next developer’s change to be propagated. The breadth of the model state trees ranges from two to 176.

The time deviations in the column time from Table 4 are explained by the applied consistency rules and the resulting inconsistency. On smaller models (# Model Elements), the change propagation might take longer than on larger models since the inconsistencies can be more dependent on each other, i.e., repairing one inconsistency causes more inconsistencies. Furthermore, the complexity of the inconsistency plays also a major role, i.e., the amount of model elements affected by the inconsistency and the amount of OCL expressions used from the consistency rule. One complex inconsistency can have more impact on the runtime than many simple inconsistencies.

#### RQ3

To assess the quality of our generated repair sequences, we applied the same strategy (explained in **RQ1**) to the two versioned models (pro11 and fullAdder) from GitHub, and compared the repair sequences applied manually by the developers to our computed repair trees. We were able to generate repair sequences consisting of all the repairs the developers also have applied in their manual repair from version 1 to version 2 in *pro11* and *fullAdder*. Additional 20 alternative repairs were provided per change on average. This shows that our change propagation approach is useful in computing repair sequences that developers actually applied manually. However, the limitation of those two models is, that we did not know the initial developer’s change and we do not know how the actual change propagation process was executed. For example, how did the developers come up with possible repairs for the inconsistencies? How long did it take to propagate their change and how many modeling experience do they have? To gain more evidence we conducted an experiment to investigate change propagation based on model inconsistency repair.

The experiment was performed with students who had to propagate a set of changes and repair caused inconsistencies. For each inconsistency, a set of repairs was provided. In total, subjects had to repair four inconsistencies from two consistency rules with 46 repairs in total (minimum of 8 repairs and a maximum of 14 repairs per inconsistency).

The premise was always the same: The subjects were given inconsistencies with their possible repairs, and they were asked to choose one repair per inconsistency which propagates the initial change causing the inconsistency. The experiment thus set the initial condition to explore how developers propagate initial changes by repairing inconsistencies. Thus, we can compare the results with our automated change propagation approach.

In the experiment 22 students participated, all master-level computer science students at our university. Their professional programming/developer experience was an average of 2 Years and 3 months. We used a medium sized UML model consisting of Class, Sequence, and State chart diagrams with the consistency rules from Table 1.

In the experiment, every student came up with one repair sequence for each of the four provided changes. Those sequences contained on average four repairs consisting of renaming, removing already existing model elements or adding new model elements. Most of the applied repairs were renames of model elements. Only two students focused on adding new model elements (e.g., adding an operation or message). Also, every repair sequence from the students was unique, since everyone chose at least one repair the others did not. Thus we can compare our change propagation with 22 different repair sequences.

Our approach was able to generate all repair sequences from all students and additionally suggested 15 alternative repair sequences. For example, one change was to propagate a rename of a transition (similar to the one from Sect. 2). One student chose to propagate the change by first adding this operation to the corresponding class and then renaming the corresponding message in the sequence diagram. Another student chose to rename first a message from the class’ corresponding lifeline, then adding this operation to the transition’s corresponding class. Our computed model state tree in this case provided both applied repair sequences.

This further shows the usefulness of our approach to developers in repairing inconsistencies with change propagation. The time to generate the propagation tree for our four changes was 300ms (75ms on average per change). The average time for the students to propagate all four changes 15 minutes (3.75 minutes on average per change). Using our approach would thus reduce the time significantly needed to propagate a change in a consistent manner.

## Threats to validity

In this section we discuss internal, external and conclusion threats to validity according to Wohlin et al. [52]

### Internal validity

The internal threats to validity are centered on the used repairs in our evaluation. As explained in Sect. 5 we needed models where developers modified the model, and those modifications lead to inconsistencies. We did not find any models with this constraint. The threat to validity here is that we were not able to propagate changes that were directly caused by a developer. However, interpreting already existing repairs as developer changes is still a valid strategy, since those repairs have been caused by a developer change in the past, and those repairs are a direct consequence of this change. Repairs are also model changes with the same implications as a change made by a developer.

To further mitigate this threat we conducted an experiment (explained in **RQ3** from Sect. 5) with 22 students each having an average professional experience of two years to three months. In this experiment, we provided a model and repairs for inconsistencies. The threat to validity here is that this model might be biased in a way that favors our approach. To mitigate this threat we used one of our models from industry (VOD) and changed the names of some operations, messages, etc. Also we provided the repairs for arising inconsistencies. To make sure we do not miss any, we performed a consistency check with our consistency analyzing tool [40, 42] that provided all possible repairs.

Furthermore, the experiment only investigated the usefulness of our approach. We did not record the time it takes for the students to propagate multiple repair sequences, or choose from a list of repair sequences our approach generated. We are confident, that this threat is acceptable, since selecting from an already existing list is easier than creating the list manually, and then selecting a repair sequence.

### External validity

We implemented our approach for UML and OCL, although we are confident that the generation of model state trees and repair sequences is also applicable to other modeling and constraint languages, we cannot generalize our results to all modeling constraint languages. However, the only requirement to apply our approach to other domains, is to detect inconsistencies and compute concrete repairs to fix them. In future work, we plan to evaluate on other modeling and constraint languages as well.

### Conclusion validity

Our evaluation gives promising results (quantitatively and qualitatively), demonstrating that our repair tree generation algorithm is very fast and reduces the amount of work a developer would have to perform drastically. The results in our case studies indicate that we are not only able to propagate changes the same way a developer would have had performed the propagation, but we also suggest additional correct propagation strategies. Only 22 students participated in our experiment which is not enough to gain statistical evidence. However, with those 22 students we were able to observe that our approach is indeed able to perform useful change propagation by providing the used repair sequences. To have more evident results, we plan to evaluate on more versioned models.

## Related work

Model evolution has gained significant momentum in the recent years. Common evolution types range from co-evolution of meta-models, consistency rules, transformation rules, etc. They all have in common that a developer change creates some kind of inconsistency, which has to be repaired in order to propagate this change.

UML model refactoring [33] can also be seen close to our work. However, model refactoring is performed on consistent model states while keeping the model consistency, i.e., no inconsistencies before or after refactoring. Our approach in contrast is able to perform the change propagation from inconsistent model states to consistent model states. Our work is rather complementary by repairing possible inconsistencies caused by refactoring or any model change.

In this section, we present and discuss approaches that are closest to ours.

**Inconsistency checking and repair:** Our approach relies on detecting inconsistencies and repairing them to be able to create new model states and propagate a developer change to impacted model elements. Briand et al. [1] proposed an approach to check UML consistency by applying an impact analysis to identify consistencies in UML models. König and Diskin [21] proposed an algorithm for consistency checking on inter-related models to reduce cost of inconsistency detection due to model merging. There is also approaches that rely on formal methods to detect inconsistencies (e.g., [2, 19, 46]). Moreover, Jongeling [16] proposes to detect inconsistencies and report on how to live with them in an industrial case study. In [17] they further allow to detect inconsistencies for heterogeneous models. Tröls et al. [50] also propose to detect inconsistencies over different kinds of artifacts and not just models. However, those approaches do not propose repairs.

All other approaches that provide repairs may be used as input for our approach to generate concrete repairs. For instance, Xiong et al., Reder et al. and Jackson et al. use a very similar notation of abstract repairs [15, 40, 41, 54]). Those abstract repairs can then be used to generate concrete repairs based on already existing model information (including the developer change) [23].

Our approach utilizes a similar method used by Reder et al. and Kretschmer et al., to detect inconsistencies [42] and generate possible concrete repairs to fix them [23]. Furthermore, Khelladi et al. propose an approach to rank repairs for a single inconsistency based on their side effects on the model, and analyze possible cycles of negative side effect [20]. The difference between those approaches and our approach is that we use the inconsistencies and repairs information to propagate developer changes to a consistent model state. Reder et al., Kretschmer et al. and Khelladi et al. do not consider developer changes and multiple model states for change propagation.

Approaches which use probabilistic generators to derive concrete repairs are not suited for a developer change propagation, since they might not consider developer created values or overwrite them during the generation process [13, 34]. Also they might not provide all possible solutions for a concrete repair [13, 28].

Puissant et al. [39] proposed a planning technique to generate repair plans for inconsistencies while aiming at a fast computation of repairs without assessing the relevance of the repair plans. Taentzer et al. [49] proposed to repair inconsistent models w.r.t. their metamodels. They relied on the model change history which helped in reducing the amount of possible repairs. Similarly, Ohrndorf et al. [36] use an initial change to propose repairs for arising inconsistencies. In contrast to those approaches, we propagate a change further than one repair step, addressing possibly arising inconsistencies.

In summary, the only requirement for other approaches to be used is that they provide a repair mechanism for inconsistencies which takes also the developer change into account.

**Co-evolution:** In model co-evolution, there exists a large body of work on how to detect, resolve and propagate changes to either the model or metamodel [12, 38]. We discuss some of the most recent work in this section. Kessentini et. al. propose to co-evolve models based on changes on the metamodel automatically [18]. They view this process as a multi objective optimization problem and use a specialized algorithm to propose a conflict resolution with the minimal amount of inconsistencies, changes to the model, and information loss. Furthermore, Mantz et al. propose to analyze metamodel to model consistency based on graph data structures and use coupled graph transformation as their co-evolutions [29].

In contrast to those approaches, we do not consider changes in the metamodel, but we only consider changes in the model itself. Furthermore, we propagate those changes only within the model w.r.t. its consistency. Co-evolution is mainly interested in creating new versions of metamodels or models based on changes in one or the other.

**Change propagation:** Semerath et al. propose to propagate a changes in a view model to model instances [44, 45]. The challenge here is, to trace back the change in the abstract view to the model that involves complex logic analysis, which is done using SAT solvers. In contrast to their work, we only consider changes applied to the model, and propagate those changes based on the model’s consistency. There exist also many approaches dealing with bidirectional model transformations with uncertainties [7, 8, 27]. However, those approaches try to synchronize changes between two models and only consider the consistency between themselves.

**Program repair:** In program repair, the literature tries to suggest repairs for malformed programs. Muşlu et al. [35] proposed to detect consequences of the code quick fixes but without exploring propagation to repair the program. Steimann et al., propose an approach for finding fixes for malformed programs based on constraint attribute grammars [47, 48]. This approach proposes deep fixing which generates repairs that avoid new violations. Cuadrado et al. [4] proposed to compute quick fixes for ATL transformations. They also proposed to detect side effects for each quick fix. In contrast to our approach, these works do not consider initial developer changes to automatically propagate, and tries to avoid negative side effects at all. However, our approach uses negative side effects to propagate a change, which is helpful in cases where negative side effects are unavoidable.

Martinez et al., propose Astor a framework for automated program repair [30]. It defines extension points to which users can attach already existing approaches for code transformations, search space navigation and validation candidate solutions. Astor generates program variants and generates patches/fixes for the original program. In this context, many approaches exists that compute patches for program failures, such as [24, 26, 31, 43, 53] The patches are then validated if they are indeed able to fix their corresponding bug. Other approaches propose to use mutation, to mine or to learn the patches, such as [3, 10, 22, 25] In contrast to those works, our approach is not a framework for program bug fixes. We propagate changes based on their influence of a model’s consistency. Furthermore, our approach is based on consistency rules and a metamodel. The metamodel enables our approach to be used for different domains, e.g., software models, formal specifications, etc.

The novelty of our approach is that we explore the chain of consequences of initial changes made by developers, i.e., inconsistencies caused. Based on that knowledge we propose repair sequences a developer can use to continue these initial changes. In essence propagating the initial changes to other parts of the model until it is consistent again. Many existing approaches deal with suggesting repairs for inconsistencies. However, they do not investigate the meaning of sequences of repairs-a recursible exploration of non-contradictory changes. In that regards, other approaches do not take under consideration earlier changes made by developers and ongoing consequences.

To the best of our knowledge, there exists no approach which performs change propagation of developer changes to repair their caused model inconsistencies in depth.

## Conclusion and future work

This paper presented a novel approach for automatically propagating developer changes. Our approach utilized model state trees to determine possible propagation strategies (i.e., repair sequences) to propagate the change to a consistent model state. The approach first detects inconsistencies in the model caused by the initial developer’s changes, and generates repairs to fix them. It then executes those repairs to generate new model states and detects new caused inconsistencies in that state. This process is repeated recursively until no more inconsistencies are caused and the change has been propagated. We refer to this as consistent change propagation. As the last step we analyze the model state tree and present repair sequences which guide the developer in propagating the initial change.

Our evaluation applied 20 consistency rules to 18 models. To check the relevance of our repair sequences, we used 2 versioned models from GitHub and performed an experiment with 22 students. We showed that our approach provides repair sequences that developers also have applied and additionally suggested alternative repair sequences. Furthermore, we have shown on larger models that our approach is scalable. We were able to propagate 176 developer changes within three seconds on average. Our approach saves time and effort that would have been spent on manually propagating a change through an entire model.

For future work, we plan to further evaluate on experiments to gain more evidence on consistent change propagation. Additionally, we will apply evolutionary algorithms to propagate changes in cases where there are too many repair sequences. This might help in cases where the developer is overwhelmed by the number of repair sequences our approach suggests in the end. Finally, we will investigate other ranking heuristics for the repair sequences.

## Footnotes

1 https://figshare.com/s/e27177256a9dc10693d2
